# Histone deacetylase 3 inhibition alleviates type 2 diabetes mellitus-induced endothelial dysfunction via Nrf2

**DOI:** 10.1186/s12964-020-00681-z

**Published:** 2021-03-18

**Authors:** Shuai Huang, Gen Chen, Jia Sun, Yunjie Chen, Nan Wang, Yetong Dong, Enzhao Shen, Zhicheng Hu, Wenjie Gong, Litai Jin, Weitao Cong

**Affiliations:** 1 Zhejiang Provincial Key Laboratory of Interventional Pulmonology, The First Affiliated Hospital of Wenzhou Medical University, Wenzhou, 325000 People’s Republic of China; 2 School of Pharmaceutical Science, Wenzhou Medical University, Wenzhou, 325000 People’s Republic of China

**Keywords:** T2DM, Endothelial dysfunction, HDAC3, Nrf2

## Abstract

**Background:**

The mechanism underlying endothelial dysfunction leading to cardiovascular disease in type 2 diabetes mellitus (T2DM) remains unclear. Here, we show that inhibition of histone deacetylase 3 (HDAC3) reduced inflammation and oxidative stress by regulating nuclear factor-E2-related factor 2 (Nrf2), which mediates the expression of anti-inflammatory- and pro-survival-related genes in the vascular endothelium, thereby improving endothelial function.

**Methods:**

Nrf2 knockout (Nrf2 KO) C57BL/6 background mice, diabetic db/db mice, and control db/m mice were used to investigate the relationship between HDAC3 and Nrf2 in the endothelium in vivo. Human umbilical vein endothelial cells (HUVECs) cultured under high glucose-palmitic acid (HG-PA) conditions were used to explore the role of Kelch-like ECH-associated protein 1 (Keap1) –Nrf2–NAPDH oxidase 4 (Nox4) redox signaling in the vascular endothelium in vitro. Activity assays, immunofluorescence, western blotting, qRT-PCR, and immunoprecipitation assays were used to examine the effect of HDAC3 inhibition on inflammation, reactive oxygen species (ROS) production, and endothelial impairment, as well as the activity of Nrf2-related molecules.

**Results:**

HDAC3 activity, but not its expression, was increased in db/db mice. This resulted in de-endothelialization and increased oxidative stress and pro-inflammatory marker expression in cells treated with the HDAC3 inhibitor RGFP966, which activated Nrf2 signaling. HDAC3 silencing decreased ROS production, inflammation, and damage-associated tube formation in HG-PA-treated HUVECs. The underlying mechanism involved the Keap1–Nrf2–Nox4 signaling pathway.

**Conclusion:**

The results of this study suggest the potential of HDAC3 as a therapeutic target for the treatment of endothelial dysfunction in T2DM.

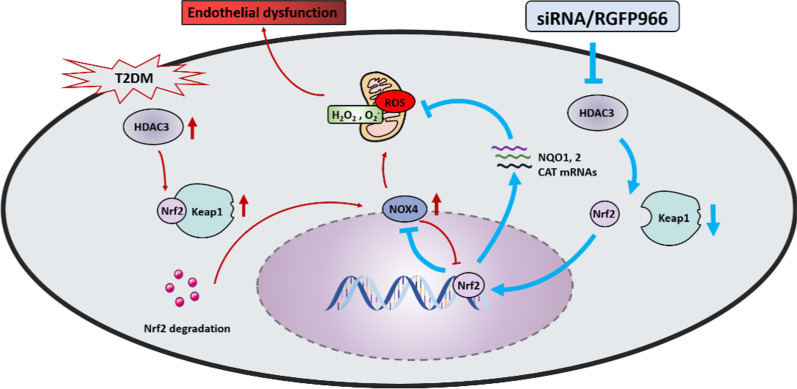

**Video Abstract**

**Supplementary Information:**

The online version contains supplementary material available at 10.1186/s12964-020-00681-z.

## Background

Type 2 diabetes mellitus (T2DM) is a chronic metabolic disorder characterized by reduced insulin action, increased hepatic glucose production, and the development of diabetic vascular complications [1]. Globally, approximately 425 million people are affected by T2DM [2]. Diabetic vasculopathy (DV) is a long-term complication of diabetes [3]. Endothelial dysfunction is the first step in the development of DV, and is associated with increased oxidative stress and inflammation [4, 5]. Developing effective methods to prevent or retard the progression of DV associated with oxidative stress is critical. Aberrant production of reactive oxygen species (ROS) activates nuclear factor-κB (NF-κB) [6], which plays a critical regulatory role in the release of pro-inflammatory cytokines, such as interleukin (IL)-1β, IL6, IL8, and tumor necrosis factor-alpha (TNF-α), as well as vascular cell adhesion molecule-1 (VCAM-1) and intracellular adhesion molecule-1 (ICAM-1) [7]. ROS are produced continuously as natural byproducts of the normal metabolism of oxygen and play important roles in redox signaling [8, 9]. Cells have evolved highly regulated endogenous antioxidant defense systems to counteract ROS overproduction, and the ability of endothelial cells to react to diabetic-like conditions by upregulating antioxidant responses has been investigated [10, 11].

Nuclear factor-E2-related factor 2 (Nrf2) is a redox-sensitive master regulatory transcription factor that plays a protective role in endothelial cells and resides in the cytoplasm bound to Kelch-like ECH-associated protein 1 (Keap1) under normal conditions [12]. Upon Keap1 destabilization or downregulation, the Keap1–Nrf2 complex is disrupted, resulting in the failure of Keap1–Nrf2 binding and nuclear translocation of Nrf2. In the nucleus, Nrf2 binds to antioxidant response elements and regulates the expression of genes encoding phase II detoxifying enzymes such as NAD(P)H dehydrogenase quinone 1, 2 (NQO1, NQO2), superoxide dismutase 2 (SOD2), heme oxygenase 1 (HO1), and catalase (CAT). This results in the production of antioxidants that act as scavengers for diabetes-induced free radicals and alleviate DV [13, 14].

A major defense against vascular injury is endothelial nitric oxide synthase (eNOS), which generates nitric oxide (NO) in the presence of optimal concentrations of the substrate L-arginine and the cofactor (6*R*)-5,6,7,8-tetrahydrobiopterin [15, 16]. Progressive vasculopathy is associated with NO deficiency caused by eNOS dysfunction [17, 18], a phenomenon referred to as “uncoupling”. Uncoupling of eNOS results in decreased bioavailability of NO as well as increased generation of ROS [19, 20]. However, the mechanisms by which eNOS is inactivated in DV remain unclear.

NADPH oxidase (Nox) is an enzyme complex that catalyzes the transfer of electrons across biological membranes, resulting in ROS production [21, 22]. The Nox complex consists of two membrane-bound catalytic subunits (p22phox and a Nox protein) and several cytosolic regulatory subunits (p47phox, p67phox, p40phox, and the small GTPase Rac) [23]. Nox4 is the predominant Nox isoform in endothelial cells, and its expression is 100‐fold higher than that of Nox1, Nox2, or Nox5 [24]. Nox family members generate superoxide, whereas Nox4 produces predominantly hydrogen peroxide [25]. Because the activity of Nox4 depends mainly on its expression level [26], abnormal expression of Nox4 affects cellular proliferation and apoptosis and is associated with the development of cardiovascular pathologies [27, 28].

Histone deacetylase (HDAC)-3 is a class I HDAC that is involved in tumor development, DM, inflammation, and cardiovascular and neurodegenerative diseases [29]. Small interfering RNA (siRNA)-mediated knockdown of HDAC3 prevents the binding of transcription factor(s) and polymerase(s) to the Nox4 promoter, thereby decreasing Nox4 transcription and Nox4-mediated ROS production in human endothelial cells [30]. In addition, Nox4–Nrf2 redox imbalance is associated with vascular endothelial cell dysfunction [21]. These findings led us to hypothesize that T2DM-induced inactivation of Nrf2 signaling caused by HDAC3 inhibition may contribute to the transcriptional regulation of Nox4.

Here, we show that siRNA-mediated inhibition of HDAC3 impaired the binding of Keap1 to Nrf2 by suppressing the synthesis of Keap1, thereby increasing Nrf2 levels by preventing Nrf2 ubiquitination and proteasomal degradation. The protective effect of HDAC3 inhibition in diabetes can be partly attributed to reduced eNOS uncoupling and activation of Nrf2 signaling through the modulation of Nox4–Nrf2 redox imbalance.

## Materials and methods

### Animals

Diabetic db/db mice and their control littermates, db/m, were obtained from Jackson Laboratories (Strain: BKS.Cg-Dock7^m+/+^Lepr^db/J^). 8 week old db/db mice were separated into 4 groups and subjected to the following treatment regimens: 1) ad libitum feeding of chow diets; 2) ad libitum feeding of chow diets and vehicle injection of dimethyl sulfoxide (DMSO; Sigma-Aldrich, D8418); 3) ad libitum feeding of chow diets and RGFP966 (MCE, HY-13909) 10 mg/kg was subcutaneously injected into the mice every other day for 10 weeks[31]. RGFP966′s formula is C_21_H_19_FN_4_O which is a highly selective HDAC3 inhibitor with an IC_50_ of 80 nM and shows no inhibition to other HDACs at concentrations up to 15 μM. RGFP966 was dissolved in DMSO and diluted in 30% hydroxypropyl beta-cyclodextrin and the final DMSO concentration was no more than 1%; 4) ad libitum feeding of chow diets and GKT137831 (Selleck, S7171) by daily gavage at a dose of 60 mg/kg/day for 10 weeks[32]. The GKT137831′s formula is C_21_H_19_ClN_4_O_2_, a member of the pyrazolopyridine dione family, which is an effective dual Nox1/4 inhibitor with an IC_50_ of 110/140 nM. It is worth pointing out that Nox4 was the predominant Nox isoform in endothelial cells, and its expression was 100‐fold higher than that of Nox1, Nox2, or Nox [24] and the GKT137831 was a widely used Nox4 inhibitor for animal administration [33, 34] as there is no specific Nox4 pharmacological inhibitor until now.

Additionally, mice with global Nrf2 knockout (Nrf2 KO) and their wild-type (WT) C57BL/6 control mice were purchased from Jackson laboratory. C57BL/6 mice were obtained from Model Animal Research Center of Nanjing University. Nrf2 KO mice with the T2DM background were established by feeding a high fat diet and by intraperitoneal streptozotocin injection (STZ) (Sigma-Aldrich, V900890). HFD (Medicience Ltd., Jiangsu, China, 21.9 kJ/g, 60% of energy as fat; MD12033), a month after exposure to the diets, the HFD-fed mice were administrated with 40 mg/kg/d STZ by intraperitoneal injection after overnight fasting for 5 consecutive days and normal diet mice were received the same volume of citrate buffer[35]. To assess the role of Nrf2 in T2DM, The randomized animal division results in 3 treatment groups in total. (1) HFD-STZ-induced mice were administrated with same volume of vehicle for 10 weeks. (2) HFD-STZ-induced T2DM mice were administrated with RGFP966 10 mg/kg subcutaneous injection for 10 weeks. (3) Nrf2 KO with HFD-STZ-induced T2DM mice were administrated with RGFP966 10 mg/kg subcutaneous injection for 10 weeks. After treatment, corresponding analyses were performed. All male mice were kept in a standard laboratory condition of temperature 21 ± 2℃, relative humidity 50 ± 15%, 12 h light-darkness cycles, with water and food available ad libitum. All animal experiments and methods performed in this study followed ethical guidelines for animal studies and were approved by the Institutional Animal Care and Use Committee of Wenzhou Medical University, China.

### Cell culture

HUVECs was purchased from Lonza, Basel, Switzerland and cultured in endothelial cell growth medium-2 (Lonza, EGM-2, CC-3156 & CC-4176) supplemented with 10% FBS and 1% penicillin/streptomycin in an incubator containing 95% air and 5% CO_2_ at 37℃ until the start of experiment. Before starting the experimental procedures, the medium was removed and replaced with phenol red-free low-glucose DMEM (Gibco BRL, 11054020) supplemented with 1% calf serum (Gibco BRL, 16010159) for 12 h, and then HUVECs were treated with EGM-2 consisting of either NG (5.5 mM) or HG (33 mM)—PA (100 μM) in the presence or absence of si-*HDAC3* for 72 h; palmitic acid (Sigma-Aldrich, PA, 100 μM; P5585), NG was used as control in this study; mannitol (Sigma-Aldrich, MAN, 33 mM: 5.5 mM of glucose + 27.5 mM of D-mannitol; M4125) was served as the osmotic control for the HG-PA, pharmacological antioxidant molecules N-Acetyl-L-cysteine (Sigma-Aldrich, NAC, 2 mM; V900429) was pretreated for 2 h every day to evaluate the effect of si-*HDAC3* on the oxidative stress. Media were changed every 24 h. The NOS inhibitor L-NAME (MCE, 100 μM; HY-18729A) co-incubated with HG-PA for 72 h was used to evaluate ROS accumulation during T2DM progress in vitro. The Sulforaphane (Selleck, SFN, 10 μM; S5771) treatment for 72 h, was used to facilitate Nrf2 nuclear translocation which were used positive control to reconfirm si-*HDAC3* cytoprotection through the similar mechanism. Ubiquitination of Nrf2 were detected using immunoprecipitation after treatment with MG132 (Selleck, 5 μM; S2619) pretreatment for 10 h before si-*HDAC3* administration.

For RNA interference, cells were transfected with human HDAC3 siRNA (si*-HDAC3*) (Santa Cruz Biotechnology, sc-355380), human Nrf2 siRNA (si*-Nrf2*) (Santa Cruz Biotechnology, sc-37030), human Nox4 siRNA (si*-Nox4*) (Santa Cruz Biotechnology, sc-41586) or control scramble siRNA by Lipofectamine 3000 for 12 h in Opti-MEM. After the transfection, cells were removed to full-growth medium for another 12 h and then analyzed for further studies.

### TUNEL staining

The cells were then stained with an In Situ Cell Death Detection Kit (Roche, 11684795910) according to the manufacturer’s protocol. The stained cells were imaged with a confocal laser scanning microscope (TCS SP8, Leica). One hundred cells per field were counted and the percentage of TUNEL-positive cells was calculated.

### In vitro* angiogenesis (Tube formation) assay *[36]

The in vitro angiogenic activity of HUVECs was determined by Matrigel tube formation assay. After the experimental period described above, the HUVECs were stained with cell-permeable dye, calcein (Corning, 354216), for 30 min and replated in 24-well plates precoated with 150 μL/well growth factor-reduced Matrigel (Corning, 354234) and incubated at 37℃ in cell culture incubator. After 12 h of incubation, capillary-like tube formation was observed with a computer-assisted microscope (Thermo Fisher Scientific, EVOS). Tube formation was defined as a tube-like structure exhibiting a length four times its width. The tube length in duplicate wells was counted and averaged using ImageJ software.

### Aortic ring assays

To establish a direct action of HDAC3 on vascular, thoracic aortae from 8-week-old C57/BL6 mice were surgically isolated, cleaned and dissected into 0.5 mm rings. Rings were embedded in 1 mg/mL of type I collagen (Millipore, 08–115) in a 96-well plate as described previously [37]. When embedded, the rings were cultured in serum-free endothelial basal medium (EBM) (Lonza, CC-3121) consisting of either NG or HG-PA in the presence or absence of si-*HDAC3*. Endothelial microvessel sprouts growing out from the rings were counted during the exponential growth phase to obtain angiogenic response data. Before the regression phase, rings were fixed with 4% (w/v) paraformaldehyde for 30 min. Pictures were taken on day 12 with a computer assisted microscope (Eclipse Ni, NIKON), and the total number of branches was counted using ImageJ (National Institutes of Health).

### Isolation of mouse aortic endothelial cells

Following the instructions by the established method for aortic endothelial cells isolation in mouse [38], briefly, the thoracic aortae of mice were surgically isolated and carefully rinsed free of blood with PBS containing 1000 U/mL heparin (Selleck, S1346), and immersed in 20% FBS-DMEM (1 g/L) containing 1000 U/mL heparin. The fat and connecting tissue were quickly removed under a stereoscopic microscope. The inside of the lumen was washed with serum-free DMEM (1 g/L), filled with collagenase II (Sigma-Aldrich, 2 mg/mL; C6685), and incubated at 37℃, for 45 min. Endothelial cells were removed by flushing with 5 mL 20% FBS-DMEM (1 g/L), and collected by centrifuging at 1200 rpm, for 5 min. The cells were then suspended gently with 20% FBS-DMEM (1 g/L), and seeded in a type I collagen-coated dish. After 7 to 10 days, confluent endothelial cells were observable.

### HDAC3 activity assay

HDAC3 activity was assessed using the HDAC3 activity assay kit (Sigma, EPI004) according to the manufactures’ protocols.

### DHE staining

Dihydroethidium (DHE) (Thermo Fisher Scientific, D11347) staining was performed as previously described [39]. HUVECs cultured in different medium stained with DHE (25 μΜ) for 30 min at 37 °C, then medium was replaced by low-glucose DMEM to monitor the generation of ROS. Pictures were captured using a computer-assisted microscope.

### Immunofluorescence staining

For quantification of CD31, 3-NT or Ki67 positive area, immunofluorescence staining was performed within the aortic wall. Eight micrometer paraffin sections were cut and incubated with anti-CD31 (Abcam, ab28364) or anti-3-NT (Abcam, ab61392) or anti-Ki67 (CST, 12,075). After washing, samples for CD31 were incubated with Alexa Fluor 488-conjugated anti-rabbit IgG secondary antibody (Abcam, ab150077) at a dilution of 1:200 for 60 min at room temperature, and samples for 3-NT were incubated with Alexa Fluor 647-conjugated anti-mouse IgG secondary antibody (Abcam, ab150115) at a dilution of 1:200 for 60 min at room temperature. The anti-Ki67 and TUNEL staining have their own fluorescent label, and aortic rings were conducted the Ki67 and TUNEL staining according to protocol. Cell nuclei were labeled by DAPI. The stained aortic walls were imaged with a confocal laser scanning microscope (Leica, TCS SP8). The total tissue area and the CD31, 3-NT or Ki67-stained positive area was measured with ImageJ software. Data were expressed as percentage of positive staining area per analyzed area.

For quantification of nuclear localization of Nrf2 in HUVECs, briefly, cultured cells in six-well plates were fixed with 4% (w/v) paraformaldehyde for 30 min and permeabilized with 0.5% (v/v) Triton-X 100 (Solarbio, Beijing, China) for 10 min at 37℃. After wash, the cells were blocked in 5% (v/v) BSA for 2 h and incubated with primary rabbit antibody against Nrf2 (Abcam, ab31163) at a dilution of 1:200 at 4 °C overnight. After incubation and wash, cells were blocked with Alexa Fluor 488-conjugated anti-rabbit IgG secondary antibody (Abcam, ab150077) for 1 h. After washes with PBS, the cell nuclei were stained with DAPI for 19 min. Photo capture was performed by a confocal laser scanning microscope. Randomly selected the perspective nuclear localization of Nrf2 and DAPI cells were under the microscope.

### Western blotting and antibodies

Equal amounts of protein lysates from HUVECs was harvested and lysed by SDS-PAGE and transferred to PVDF membrane (Merck Millipore, IPVH00010), then subjected to western blotting analysis: Membranes were blocked with 5% (v/v) bovine serum albumin (BSA) in Tris-buffered saline containing 0.1% (v/v) Tween 20 (TBST) and incubated with primary antibodies overnight at 4℃. The proteins were visualized using an Image Quant LAS 4000 (GE Healthcare) system, and the secondary antibodies are: goat anti-rabbit HRP (Bio-Rad, 1706515), goat anti-mouse HRP (Bio-Rad, 1706516).

For nuclear Nrf2 accumulation assays, HUVECs were harvested and lysed to obtain cytoplasmic and nuclear lysates using the Keygen Nuclear-Cytosol Protein Extraction Kit from Nanjing KeyGen Biotech. Co., Ltd. (Nanjing, China).

The primary antibodies used to probe the membranes included Nrf2 (Santa Cruze Biotechnology, sc365949), Bax (Abcam, ab32503) and Bcl-2 (Abcam, ab59348) and GAPDH (CST, 5174), Lamin B (CST, 12,586), Keap1 (CST, 4678), c-Caspase-3 (CST, 9661), HDAC3 (CST, 85057), eNOS (CST, 32027), acetylated-lysine (CST, 9441), 3-NT (Millipore, 05–233), Nox4 (Origene, TA349083).

### Assay of eNOS dimer/monomer

SDS-resistant eNOS dimers and monomers were assayed by using low-temperature SDS-PAGE as described previously [40]. Briefly, after being washed twice with ice-cold phosphate-buffered saline, D-glucose-treated HUVECs were lysed as described above, and protein lysates were mixed with loading buffer and loaded on gels without boiling. Proteins were separated with low-temperature SDS-PAGE under reducing conditions (with -mercaptoethanol). Gels and buffers were kept at 4℃ during the whole procedure.

### Co-immunoprecipitation

Cell lysates with 500 μg proteins were incubated with 20 μL PureProteome™ Protein A/G Mix Magnetic Beads (Merck Millipore, LSKMAGAG10) and appropriate primary antibodies at 4℃ overnight according to the manufacturer’s protocols. The supernatants of the immunoprecipitates were subjected to immunoblotting for the detection of protein–protein interaction.

### RNA extraction and qRT-PCR

Total RNA from HUVECs were isolated by Trizol reagent (BioTeke, RP1001) and reversely transcribed to cDNA using SuperScriptIII (Vazyme, R223-01). qRT-PCR was performed with products from Thermo fisher scientific Inc. (A31667) on a QuantStudio™ 3 Real-Time PCR Detection System. Glyceraldehyde-3- phosphate dehydrogenase (GAPDH) was used as a reference gene. The relative expression levels of mRNA were quantified using the ΔΔCt method. The specific primers are showed in Additional file 1: table 1.

### Statistical analysis

Data were analyzed by GraphPad Prism 5.0 and results were expressed as mean ± standard error of the mean (S.E.M.). Differences of each sample were evaluated using the unpaired Student's two-tailed t-test or analysis of variance (ANOVA). A value of *P < 0.05 was considered significant. All experiments were repeated at least three times.

## Results

### *HDAC3 inhibition rescues endothelial injury caused by T2DM *in vivo* and *in vitro

**Fig. 1:**
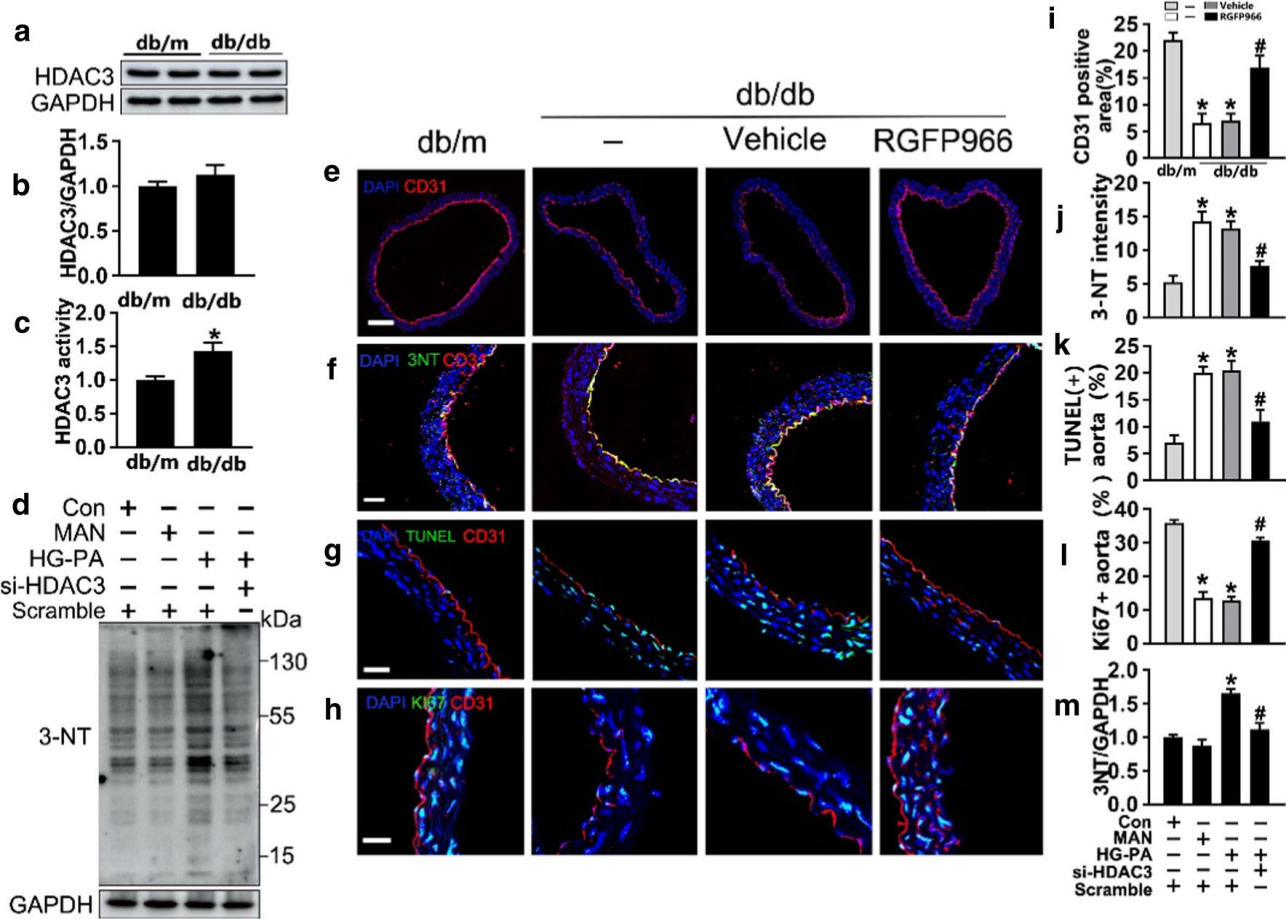
HDAC3 activity and its inhibition are related to *endothelial function* in T2DM. **a, b** The immunoblotting and quantitative analysis of HDAC3 protein level relative to GAPDH protein levels in the endothelial cells isolated from db/m mice or db/db mice. Values displayed are means ± SEM (n = 5). **c** Measurements of deacetylase activity of HDAC3 in different groups of endothelial cells isolated from db/m mice or db/db mice. **d** Levels of the oxidative damage marker 3-NT in HUVECs were detected by Western blot. **e** Representative immunofluorescence with CD31 from db/m mice, db/db mice, and RGFP966 or vehicle treated (subcutaneously, 10 mg/kg) db/db mice aorta tissue sections. The red area represented endothelium and the nucleus was blue. Scale bars: 200 μm. **f** Representative confocal images of oxidative damage marker 3-NT in aortal vascular endothelium. The red area represented endothelium, the green area represents 3-NT positive staining and the nucleus was blue. Scale bars: 40 μm. **g** Representative confocal images of apoptosis in aortal vascular endothelium. The red area represented endothelium, the green area represents TUNEL positive staining and the nucleus was blue. Scale bars: 40 μm. **h** The presence of immunofluorescence with CD31 and Ki67 of aortal vascular endothelium, scale bars: 20 μm, the green area represents Ki67 positive staining and the nucleus was blue. **i–l** Quantification of the number of CD31 positive area staining (**e**), the number of 3-NT staining (**f**), TUNEL + cells (**g**), the proportion of Ki67 positive staining (**h**). **m** The quantitative analysis of 3-NT protein immunoblotting, values displayed are means ± SEM (n = 4). Significance (**c, i, j, k, l**): *P < 0.05 vs. db/m mice; #*P* < 0.05 vs. db/db mice or vehicle treated db/db mice. Significance (**m**): *P < 0.05 vs. Con or MAN in scrambled HUVECs; #*P* < 0.05 vs. HG-PA in scrambled HUVECs. All above results in graphs from western blot were normalized to the first group

The role of HDAC3 in the endothelium was investigated using endothelial cells isolated from aorta of diabetic db/db and nondiabetic db/m mice. HDAC3 activity, but not protein level, was significantly higher in the db/db group than in the db/m group (Fig. 1a and c). The relationship between HDAC3 and endothelial dysfunction was examined by treating mice with the specific HDAC3 inhibitor RGFP966 by subcutaneous injection at a dose of 10 mg/kg. HDAC3 inhibition significantly restored T2DM-induced de-endothelialization in diabetic mice compared with that in the vehicle-treated group, demonstrating the protective effect of HDAC3 inhibition against T2DM-induced endothelial impairment in vivo (Fig. 1e and i).

T2DM-induced endothelial dysfunction is mediated by several mechanisms, of which oxidative stress and pro-inflammatory responses originate in the endothelium. We used 3-nitrotyrosine (3-NT) as an oxidative stress marker and showed that si-*HDAC3* treatment alleviated high glucose-palmitic acid (HG-PA)-induced oxidative stress from HUVECs (Fig. 1d and m). Immunofluorescence analysis showed that 3-NT staining intensity in the aortic vascular endothelium was higher in diabetic mice than in the db/m group, and this was decreased by HDAC3 inhibition (Fig. 1f and j). Next, we analyzed the effect of RGFP966 on T2DM-induced vascular apoptosis in diabetic aortic endothelial cells. The results showed that the accumulation of apoptosis puncta in the aortic vascular endothelium (labeled with CD31) was greater in db/db mice than in the corresponding control littermates, whereas treatment with RGFP966 significantly reduced apoptosis signals compared with those in the vehicle-treated group (Fig. 1g and k). RGFP966 treatment increased the Ki67 and CD31 positive area, indicating that HDAC3 inhibition promoted vascular endothelial proliferation during diabetic vascular impairment (Fig. 1h and l).

**Fig. 2:**
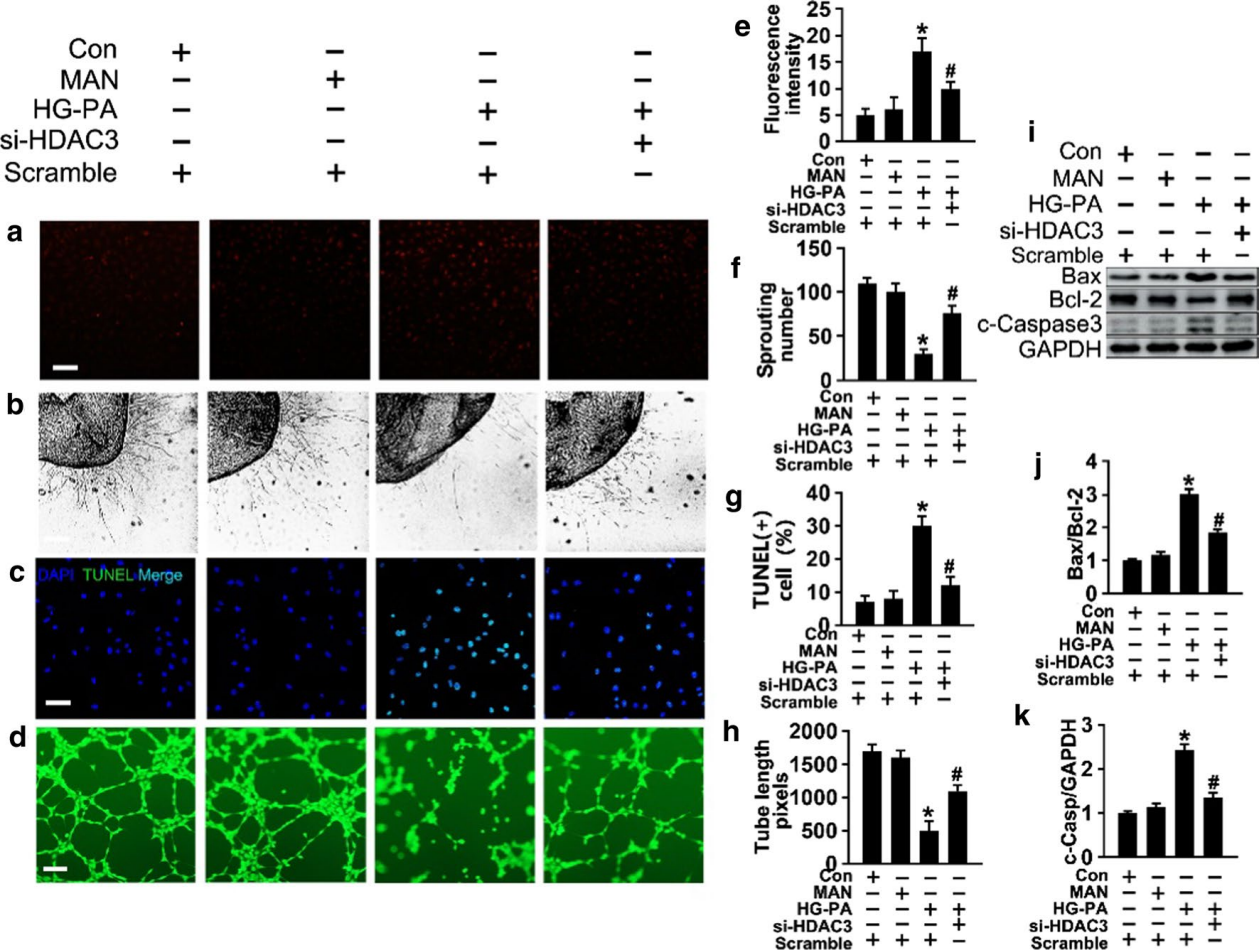
The effect of HDAC3 inhibition in T2DM–induced *endothelial dysfunction *in vitro. **a** Fluorescent images of superoxide levels in HUVECs cultured either in Con (5.5 mM of glucose), MAN (33 mM: 5.5 mM of glucose + 27.5 mM of D-mannitol) or HG (33 mM)-PA (100 μM) medium with or without si-*HDAC3* for 72 h, MAN was served as the osmotic control for the HG-PA. Superoxide was determined with the fluorescent indicator DHE, and the fluorescent intensity of DHE was observed with a computer-assisted microscope. Scale bars: 100 μm. **b** Representative images of aortic rings from db/m mice were cultured in medium. Scale bars: 200 μm. **c** TUNEL assay of HUVECs was determined. The apoptotic cells were labeled with green, and nuclei were stained with DAPI (blue). Scale bars: 100 μm. **d** Capillary-like tube formation was assessed by Matrigel angiogenesis assay in HUVECs. Scale bars: 300 μm. **e–h** Quantification of the Fluorescence intensity in (**a**), number of sprouts in (**b**), TUNEL + cells in (**c**), the tube length in (**d**). **i** Cell lysates of HUVECs were used to detect Bax, Bcl-2 as well as c-Caspase 3 protein levels by immunoblotting. **j**, **k** The quantitative analysis of each immunoblotting. The data are represented as the means ± SEM (n = 5). Significance: *P < 0.05 vs. Con or MAN in scrambled HUVECs. #*P* < 0.05 vs. HG-PA in scrambled HUVECs. All above results in graphs from western blot were normalized to the first group

Similar results were obtained in the dihydroethidium (DHE) assay, which indicated that si-*HDAC3* alleviated HG-PA-induced superoxide production (Fig. 2a and e). The HG-PA-induced increase in the number of TUNEL-positive cells (Fig. 2c and g), and the increased c-caspase 3 levels and Bax/Bcl-2 ratio (Fig. 2i–k), indicated that T2DM increased endothelial cell apoptosis, and these effects were reversed by si-*HDAC3* treatment. In the aortic ring assay, aortic rings from C57BL/6 mice were cultured in different media. Aortic rings cultured in normal medium showed a well-structured microvessel network with clearly defined tubules and regular branching. HG-PA treatment dramatically impaired sprouting, whereas HDAC3 knockdown reversed this effect (Fig. 2b and f). Tube-forming activity was also significantly impaired in HUVECs exposed to HG-PA compared with the controls, whereas treatment with si-*HDAC3* restored tube formation (Fig. 2d and h).

Next, we examined the effect of HDAC3 inhibition on eNOS uncoupling, which leads to endothelial dysfunction [41]. For this purpose, the proportion of eNOS existing as dimers or monomers in HUVECs treated with HG-PA in the presence or absence of si-*HDAC3* was determined. eNOS dimer formation and stability are critical for the production of NO by eNOS, and a decrease in the dimer-to-monomer ratio indicates eNOS uncoupling [42]. Low-temperature SDS-PAGE (without heat denaturation) of HUVECs lysates detected bands approximately twice the size of the eNOS monomer, probably corresponding to eNOS dimers. Exposure of the cells to HG-PA for 72 h significantly decreased the dimer-to-monomer ratio compared with that in the control, and this effect was markedly reversed in response to si-*HDAC3* (Additional file 1: Fig. 1E and F). Under pathological conditions, eNOS uncoupling results in increased generation of superoxide anion; therefore, we examined eNOS uncoupling-induced intracellular superoxide generation using the DHE assay and confocal microscopy. Incubation of HUVECs with HG-PA in the presence of si-*HDAC3* significantly decreased ROS production (Fig. 1a and e). The contribution of uncoupled eNOS-to-ROS production was determined by measuring superoxide generation in the presence and absence of the eNOS inhibitor NG-nitro-L-arginine methyl ester (L-NAME). Inhibition of HG-PA-induced superoxide generation by L-NAME indicates that eNOS is a source of ROS and, therefore, that the enzyme is uncoupled (Additional file 1: Fig. 1a and 1b). Collectively, these findings indicate that endothelial dysfunction caused by uncoupled eNOS was decreased by si-*HDAC3* in HUVECs exposed to HG-PA. These data provide a rationale for studying the mechanism(s) by which the enzyme is uncoupled in these cells. In addition, the results suggest HDAC3 inhibition as a potential novel approach to the treatment of T2DM-induced endothelial impairment.

### HDAC3 inhibition activates Nrf2 signaling by decreasing Keap1–Nrf2 interaction

**Fig. 3:**
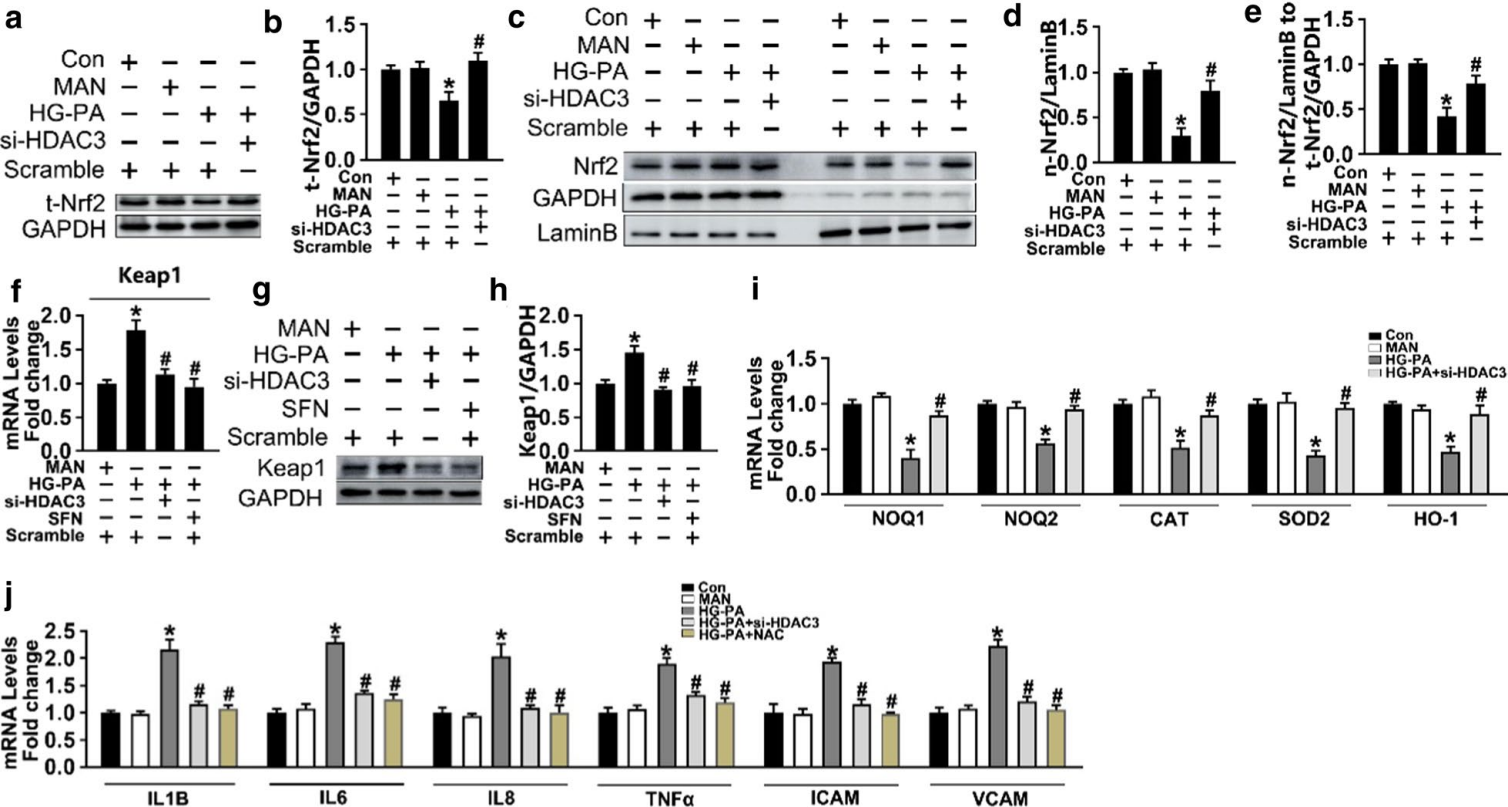
HDAC3 inhibition activates Nrf2 signaling in vitro. **a** and **c** Expression of total (T-Nrf2), cytosolic (c-Nrf2) and nuclear (n-Nrf2) protein levels of Nrf2 were detected by Western blot. **b**, **d and e** The quantitative analysis of each immunoblots. **f** The mRNA level and the quantification of Keap1 were evaluated by qRT-PCR. **g** the protein level of Keap1 were detected by western blot. **h** The quantitative analysis of (**g**). **i** The mRNA level and the quantification of the Nrf2 target gene were evaluated by qRT-PCR. **j** The mRNA level and the quantification of the NF-κB target gene were evaluated by qRT-PCR. The data are represented as the means ± SEM (n = 4). Significance: *P < 0.05 vs. Con or MAN in scrambled HUVECs; #*P* < 0.05 vs. HG-PA in scrambled HUVECs. All above results in graphs were normalized to the first group

**Fig. 4:**
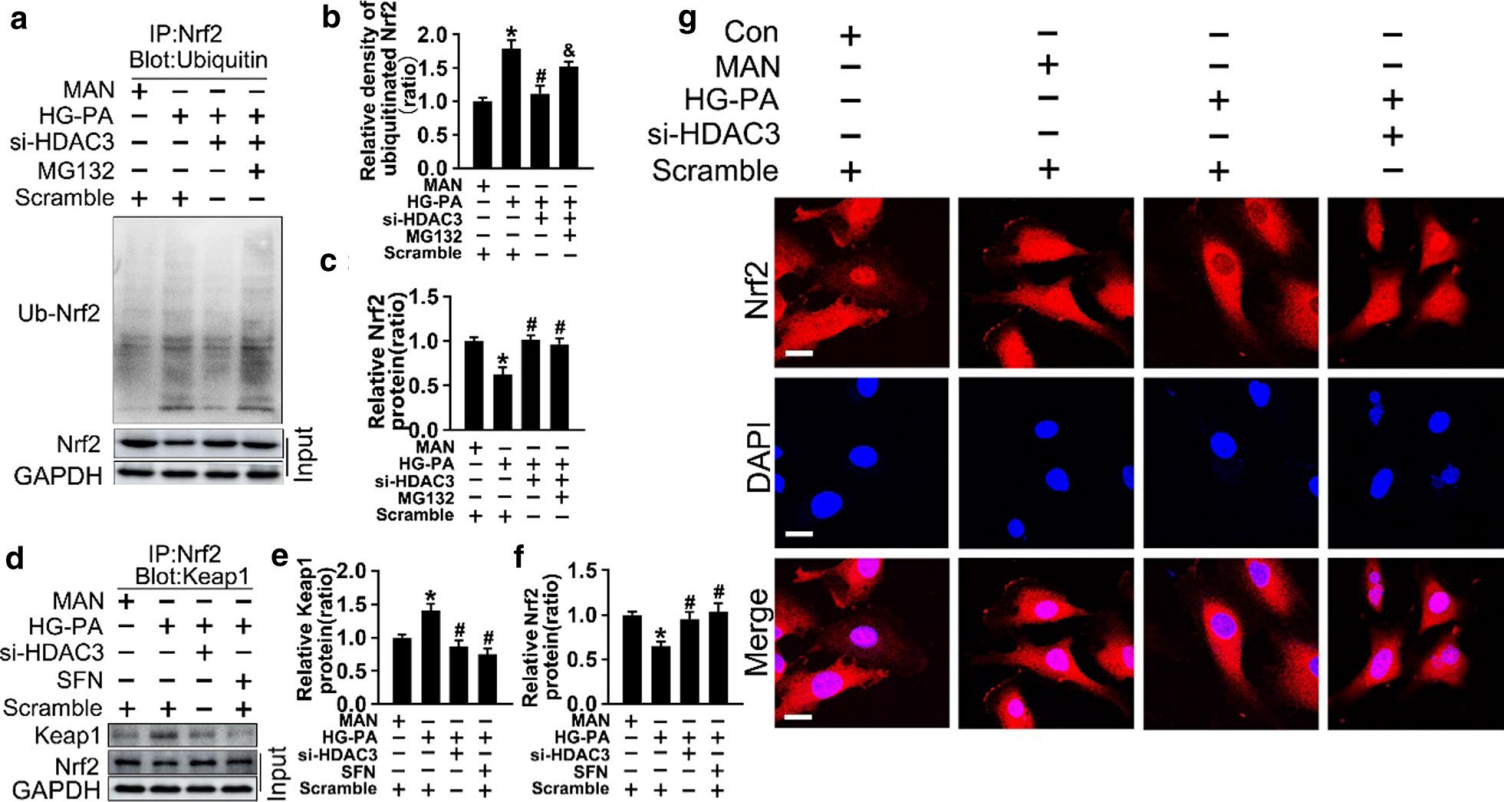
HDAC3 inhibition activates Nrf2 signaling by decreasing Keap1–Nrf2 interaction. **a** HUVECs were cultured in different condition, and the cell lysates were subjected to immunoprecipitation with Nrf2 antibody, followed by immunoblotting with indicated antibody. **b and c** The quantitative analysis of (**a**). **d** The cell lysates of HUVECs were subjected to immunoprecipitation with Nrf2 antibody, followed by immunoblotting with indicated antibody. **e and f** The quantitative analysis of (**d**). **g** Nrf2 nuclear translocation was determined in fixed cells by immunofluorescent staining in HUVECs. The red area represented Nrf2 and the nucleus was blue. Scale bars: 15 μm. The data are represented as the means ± SEM (n = 4). Significance: *P < 0.05 vs. MAN in scrambled HUVECs, #*P* < 0.05 vs. HG-PA in scrambled HUVECs, & *P* < 0.05 vs. HG-PA with si-*HDAC3*. All above results in graphs were normalized to first group

Because decreased nuclear Nrf2 signaling is associated with oxidative stress [43], we hypothesized that HDAC3 inhibition could protect against HG-PA-induced endothelial dysfunction by regulating Nrf2-modulated antioxidant gene expression. Western blot analysis of Nrf2 expression showed that HG-PA significantly downregulated nuclear and total Nrf2 in endothelial cells, whereas co-treatment with si-*HDAC3* restored Nrf2 levels (Fig. 3a–d). Assessment of the ratio of nuclear Nrf2 (n-Nrf2)/lamin B to cytoplasmic Nrf2 (c-Nrf2)/GAPDH (Fig. 3e) confirmed the results of western blotting and immunofluorescence, showing that si-*HDAC3* decreased Nrf2 nuclear translocation in endothelial cells (Fig. 4g).

qRT-PCR analysis of the mRNA levels of Nrf2 target genes showed that HG-PA downregulated NQO1, NQO2, CAT, SOD2, and HO1, and this effect was partly rescued by si-*HDAC3* treatment (Fig. 3i). T2DM-induced oxidative stress activates NF-κB, which regulates the release of pro-inflammatory cytokines such as IL1β, IL6, IL8, TNFα, VCAM-1, and ICAM-1. Consistently, the mRNA expression of NF-κB target genes was significantly higher in endothelial cells exposed to HG-PA than in those cultured in NG, and this effect was attenuated by si-*HDAC3* or the pharmacological antioxidant molecule NAC (Fig. 3j).

Next, we explored the molecular mechanism underlying the effect of HDAC3 inhibition on activating the Nrf2 pathway. Keap1 sequesters Nrf2 in the cytoplasm, restricting Nrf2 from nuclear translocation and facilitating Nrf2 degradation [44]. Western blotting and qRT-PCR results showed that si-*HDAC3* co-treatment suppressed the effect of HG-PA on upregulating Keap1 expression (Fig. 3f–h). Sulforaphane (SFN), which inactivates Keap1 and promotes Nrf2 nuclear translocation, was introduced as positive control to confirm the effect of si-*HDAC3* (Fig. 3g and h). The results indicated that HDAC3 inhibition activated Nrf2 signaling by downregulating Keap1 and facilitating Nrf2 nuclear translocation.

Nrf2 is rapidly degraded by the ubiquitin–proteasome system under homeostatic conditions [45]. To determine whether the increase in Nrf2 caused by HDAC3 inhibition was associated with the suppression of Nrf2 ubiquitination, Nrf2 ubiquitination was analyzed by immunoprecipitation after treatment with MG132, a proteasome and protease inhibitor, in the presence or absence of si-*HDAC3*. As shown in Fig. 4a–c, si-*HDAC3* alone or in combination with MG132 upregulated Nrf2 expression compared with that in response to HG-PA. Immunoprecipitation with anti-Nrf2 showed that HG-PA increased Nrf2 ubiquitination (lane 2) reduced by co-treatment with si-*HDAC3* (lane 3), and this effect was reversed by MG132 administration (lane 4).

Next, cell lysates were immunoprecipitated using an anti-Nrf2 antibody and immunoblotted for Keap1 to detect the Nrf2–Keap1 interaction. Total lysates (input) were evaluated by western blotting using anti-Nrf2 antibodies. The results showed that si-*HDAC3* co-treatment inhibited the Nrf2–Keap1 interaction compared with that in HG-PA-treated endothelial cells, and this effect was similar to that of SFN co-treatment in the HG-PA medium (Fig. 4d–f).

Thus, we speculate that HDAC3 inhibition activated Nrf2 signaling by inhibiting Keap1 synthesis rather than promoting Keap1 ubiquitination, which suppressed the Nrf2–Keap1 interaction-induced ubiquitination of Nrf2, facilitating Nrf2 nuclear translocation.

### Nox4 inhibition counteracts T2DM-induced endothelial dysfunction partially through the Nrf2–Nox4 loop

**Fig. 5:**
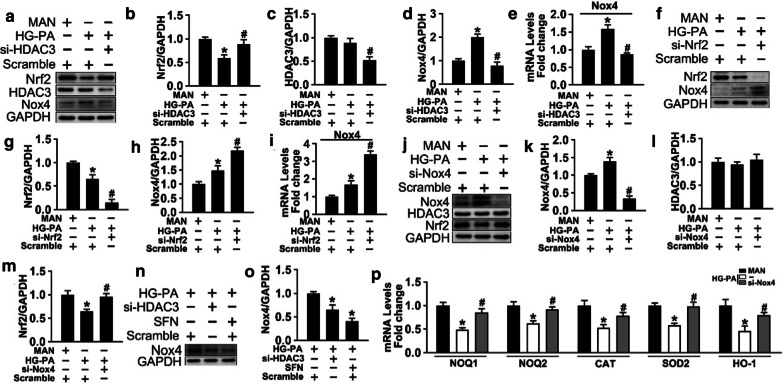
Nox4 inhibition counteracts T2DM-induced endothelial dysfunction partially through the Nrf2–Nox4 loop.* a *Cell lysates of HUVECs were used to detect indicated protein levels by immunoblotting. **b–d** The quantitative analysis of each immunoblots. **e** The mRNA level and the quantification of the Nox4 were evaluated by qRT-PCR. **f** Cell lysates of HUVECs were used to detect indicated protein levels by immunoblotting. **g** and **h** The quantitative analysis of each immunoblots. **i** The mRNA level and the quantification of the Nox4 were evaluated by qRT-PCR, which assay of HUVECs treated as indicated in (**f**). **j** Cell lysates of HUVECs were used to detect indicated protein levels by immunoblotting. **k, l and m** The quantitative analysis of each immunoblots in (**j**). **n** Cell lysates of HUVECs were subjected to immunoblot with Nox4 antibody. **o** The quantitative analysis of (**n**). **p** The mRNA level and the quantification of the Nrf2 target gene were evaluated by qRT-PCR. Data are represented as means ± SEM (n = 5). Significance: *P < 0.05 and **P < 0.01 vs. MAN in scrambled HUVECs, #*P* < 0.05 and ##*P* < 0.01 vs. HG-PA in scrambled HUVECs. All above results in graphs were normalized to first group

The Nrf2 response is associated with the induction of Nox4, namely, activated Nrf2 signaling inhibits the transcription of Nox4 in the endothelium [46]. Therefore, we examined whether Nox4–Nrf2 imbalance plays a role in the diabetic endothelium. The results showed that HG-PA treatment upregulated Nox4 transcription in HUVECs (Fig. 5a, d, and e), and this was reversed by si-HDAC3 treatment. Although the link between HDAC3 and Nrf2 was demonstrated, the relationship between Nox4 and Nrf2 remained unclear. To examine this, Nrf2 was silenced by siRNA (Fig. 5f and g). Nox4 mRNA and protein levels were higher in cells treated with si-*Nrf2* in combination with HG-PA than in those treated with HG-PA alone (Fig. 5f, h, and i). HDAC3 inhibition downregulated Nox4 to a similar level than SFN treatment, which inactivates Keap1 (Fig. 5n and o). These results indicate that HDAC3 inhibition activated Nrf2 signaling by decreasing Keap1 synthesis (Result 2), thereby downregulating Nox4. SiRNA-mediated Nox4 silencing increased Nrf2 levels (Fig. 5j and m), which was associated with an increase in the transcription of genes encoding antioxidant enzymes (Fig. 5p)*,* defining a Nrf2–Nox4 negative feedback loop.

**Fig. 6:**
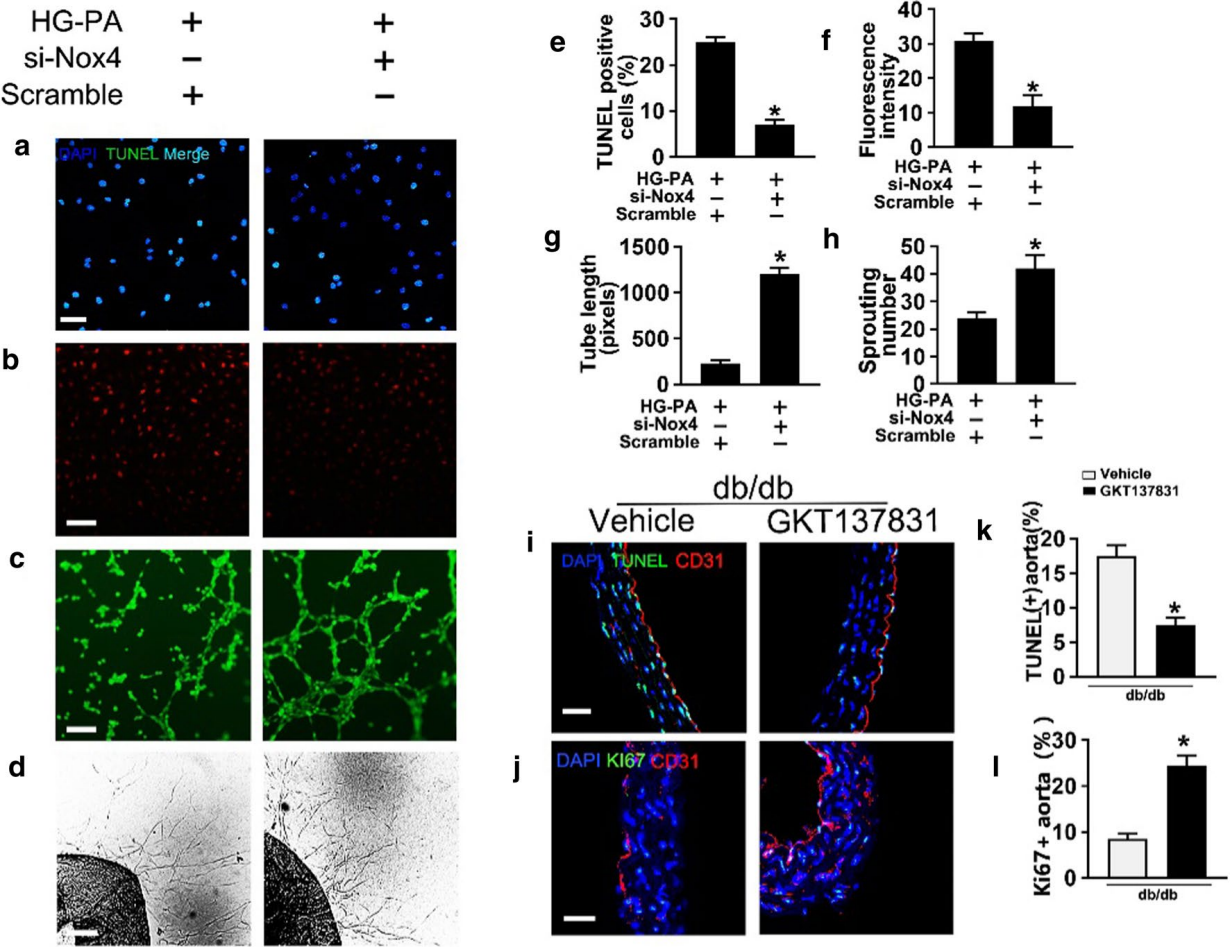
Nox4 inhibition counteracted T2DM-induced endothelial dysfunction*. a* TUNEL assay of HUVECs cultured with HG-PA alone or with si-*Nox4* for 72 h, Scale bars: 100 μm. **b** The fluorescent intensity of DHE was observed. Scale bars: 100 μm. **c** Capillary-like tube formation was assessed in HUVECs. Scale bars: 300 μm. **d** Representative images of aortic rings from db/m mice. Scale bars: 200 μm. **e–h** The quantitative analysis of TUNEL + cells in (**a**), fluorescent intensity in (**b**), the tube length in (**c**), the number of sprouts in (**d**). **i** Representative confocal images of apoptosis in aortal vascular endothelium from db/db mice or db/db mice gavaged at a dose of 60 mg/kg/day GKT137831. Scale bars: 40 μm. **J** The presence of immunofluorescence with CD31 and Ki67 of aortal vascular endothelium. Scale bars: 20 μm. **k** and **l** The quantitative analysis of TUNEL + cells and Ki67 of aortal vascular endothelium from db/db mice and GKT137831 treated db/db mice aorta tissue sections. Data are represented as means ± SEM (n = 5). Significance (**e**–**h**): *P < 0.05 vs. HG-PA in scrambled HUVECs. Significance (**k** and **l**): *P < 0.05 vs. vehicle-treated db/db mice. **j** Representative confocal images of oxidative damage marker 3-NT in aortal vascular endothelium. Scale bars: 40 μm. **k** The presence of immunofluorescence with CD31 and Ki67 of aortal vascular endothelium. Scale bars: 20 μm. **l** The quantitative analysis of TUNEL + cells from db/db mice and GKT137831 treated db/db mice aorta tissue sections. Data are represented as means ± SEM (n = 5). Significance (**e**–**h**): *P < 0.05 vs. HG-PA in scrambled HUVECs. Significance (**k** and **l**): *P < 0.05 vs. vehicle-treated db/db mice

The results of TUNEL (Fig. 6a and e), DHE (Fig. 6b and f), tube formation (Fig. 6c and g), and aortic ring assays (Fig. 6d and h) demonstrated that downregulation of Nox4 alleviated T2DM-induced endothelial dysfunction. In vivo, db/db mice treated with the Nox4 inhibitor GKT137831 showed significantly reduced apoptosis in the aortic vascular endothelium (Fig. 6i and k) and attenuated T2DM-induced de-endothelialization compared with the vehicle-treated group. The results of the Ki67 assay (Fig. 6j and l) showed that Nox4 knockdown significantly improved vascular endothelial proliferation during diabetic vascular impairment. Taken together, the results indicate that downregulation of Nox4 by HDAC3 inhibition protected against T2DM-induced endothelial dysfunction through a mechanism involving Nrf2 and the activation of the Nrf2–Nox4 negative feedback loop*.*

### Knockdown of Nrf2 abolishes the HDAC3 inhibition-induced protective effect in T2DM

**Fig. 7:**
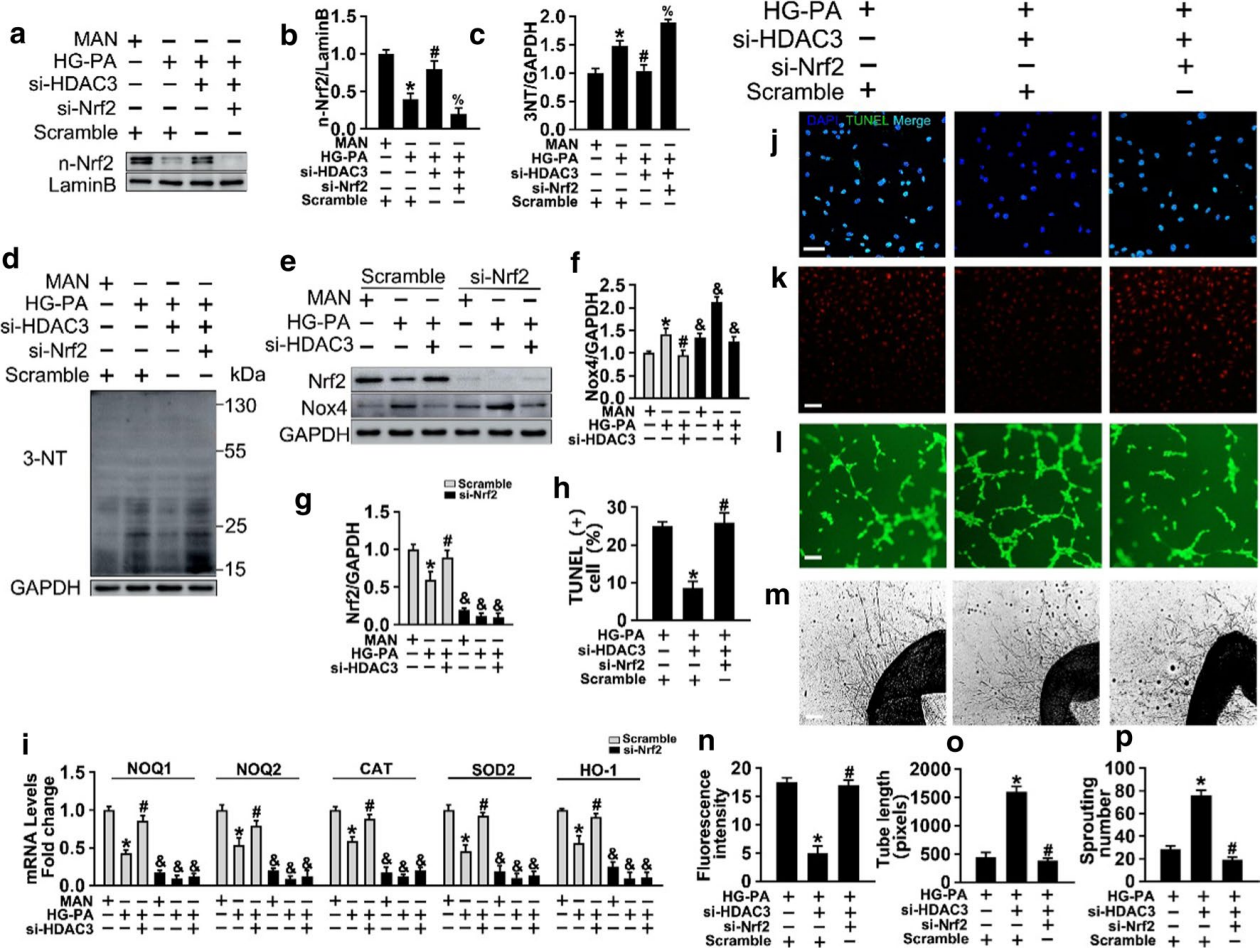
Knockdown of Nrf2 abolishes HDAC3 inhibition-induced protective effect in vitro*.* **a** HUVECs were transduced with si-*Nrf2* and a scrambled sequence (scramble) respectively. Protein expression of nuclear accumulation of Nrf2 in HUVECs after transfecting with siRNA assayed by Western blot. **b, c** The quantitative analysis of nuclear accumulation of Nrf2 and 3-NT immunoblots. **d** Levels of the oxidative damage marker 3-NT in HUVECs was detected by Western blot. **e** HUVECs were transduced with si-*Nrf2*, scrambled sequence combined with si-*HDAC3* or not. **f**, **g** The quantitative analysis of Nrf2 and Nox4 were evaluated. **h** Quantification of the TUNEL + cells in (**j**). **i** The mRNA level and the quantification of the Nrf2 target gene were evaluated by qRT-PCR. Data are represented as mean ± SEM (n = 5). Significance: *P < 0.05 vs. respective MAN in scrambled HUVECs, #*P* < 0.05 vs. respective HG-PA in scrambled HUVECs, % *P* < 0.05 vs. respective HG-PA with si-*HDAC3* cultured HUVECs, & *P* < 0.05 vs. scrambled HUVECs with the same treatment. All above results in graphs (**b**), (**c**), (**f**), (**g**), (**i**) were normalized to first group. **j** TUNEL assay of HUVECs. Scale bars: 100 μm. **k** Superoxide was determined with DHE. Scale bars: 100 μm. **l** Capillary-like tube formation was assessed. Scale bars: 300 μm. **m** Representative confocal images of aortic rings. Scale bars: 400 μm. Values displayed are means ± SEM (n = 5). Significance (**h**), (**n**), (**o**), (**p**): *P < 0.05 vs. db/db mice, #*P* < 0./05 vs. RGFP966 treated db/db mice

**Fig. 8:**
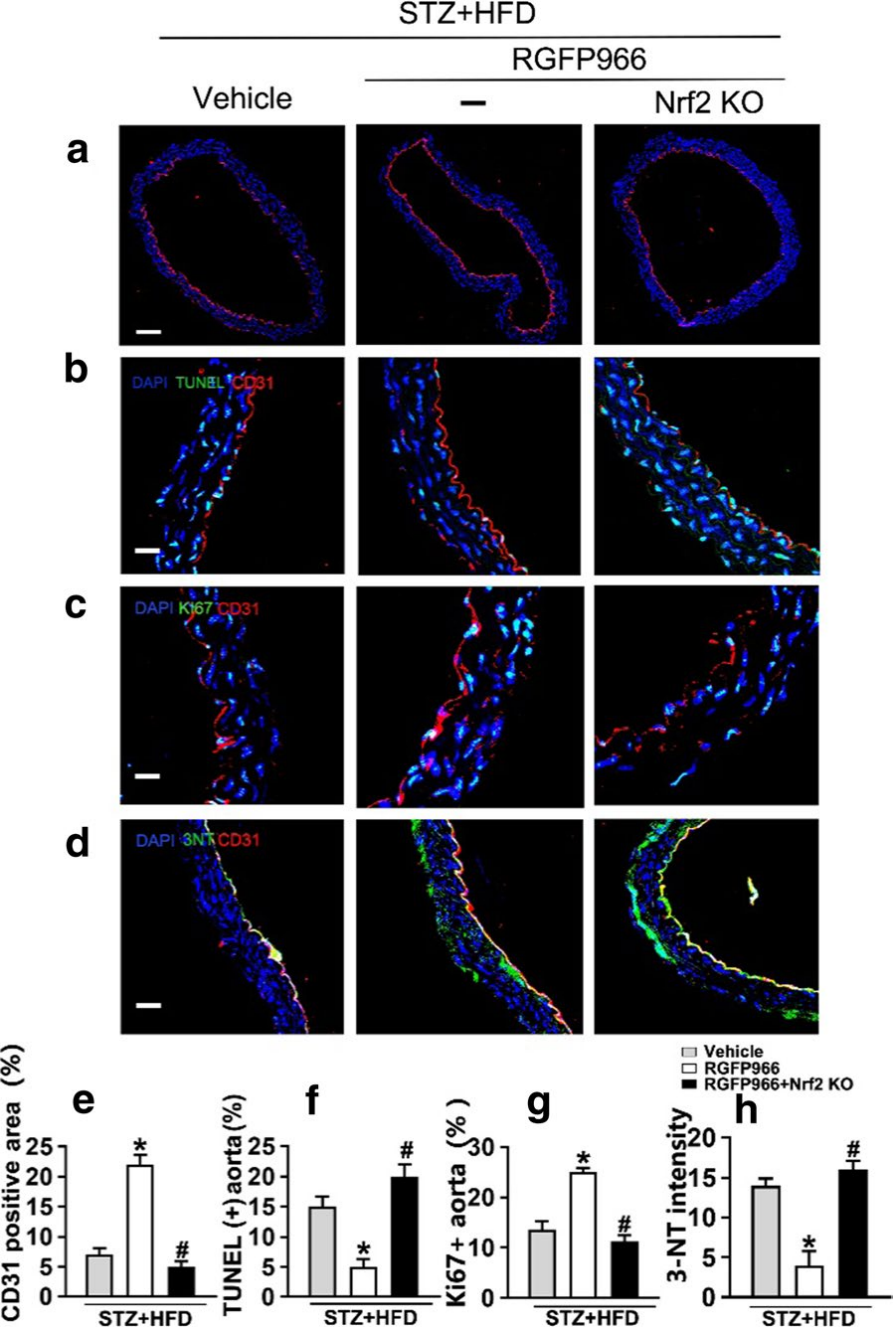
Knockdown of Nrf2 abolishes the HDAC3 inhibition-induced protective effect in vivo. **a** Representative immunofluorescence with CD31 from vehicle treated T2DM mice (fed by STZ and HFD), RGFP966 treated (10 mg/kg) T2DM mice or Nrf2 knockout mice with RGFP966 treated T2DM mice aorta tissue sections. Scale bars: 200 μm. **b** Representative confocal images of apoptosis in aortal vascular endothelium. Scale bars: 40 μm. **c** The presence of immunofluorescence with CD31 and Ki67 of aortal vascular endothelium. Scale bars: 20 μm. **d** Representative confocal images of oxidative damage marker 3-NT in aortal vascular endothelium. Scale bars: 40 μm. **e–h** Quantification of the CD31 positive area in (**a**), TUNEL + cells in (**b**), the proportion of Ki67 positive area in (**c**), the number of 3-NT staining in (**d**) respectively. Values displayed are means ± SEM (n = 5). Significance (**e–h**): *P < 0.05 vs. db/db mice, #*P* < 0.05 vs. RGFP966 treated db/db mice

To confirm the role of Nrf2 in the HDAC3 inhibition-mediated protective effect on diabetes-induced endothelial injury, si-*HDAC3*-treated HUVECs were incubated with HG-PA in the presence or absence of si-*Nrf2*. The results showed that si-*Nrf2* abolished the si-*HDAC3*-induced nuclear accumulation of Nrf2 (Fig. 7a and b) and aggravated the si-*HDAC3*-induced the expression of Nox4 (Fig. 7e and g) compared with the scrambled HUVECs with the same treatment. Furthermore, HG-PA-induced oxidative damage detected by 3-NT was attenuated by HDAC3 inhibition, and this effect was reversed by si-*Nrf2* co-treatment (Fig. 7c and d). si-*Nrf2* eliminated the protective effect of HDAC3 inhibition on T2DM, as determined by the expression of antioxidant genes (Fig. 7i), increased apoptosis (Fig. 8j and h), increased oxidative stress (Fig. 8k and n), and aberrant tube formation (Fig. 8l and o) and vascular sprouting (Fig. 8m and p).

Nrf2 KO mice with the T2DM background were established by feeding a high fat diet and by intraperitoneal streptozotocin injection (Additional file 1: Fig. 1c and d). The results of thoracic aortic sprouting, CD31 immunofluorescence staining (Fig. 8a and e), TUNEL assay (Fig. 8b and f), Ki67 detection (Fig. 8c and g), and 3-NT staining (Fig. 8d and h) indicated that Nrf2 knockout abrogated the RGFP966-induced protective effect on T2DM-induced aortic endothelial injury. Consistent with the observations in vitro, Nrf2 in the aortic endothelium was required for HDAC3 inhibition-mediated attenuation of T2DM-induced oxidative stress, inflammation, and vasculopathy.

## Discussion

In vivo and in vitro studies have demonstrated that T2DM can progressively compromise endothelial function in association with excessive ROS production and inflammation, thereby contributing to T2DM-associated vascular complications. HDACs are epigenetic regulators that catalyze the removal of acetyl groups from lysine residues in a variety of proteins. HDACs deacetylate nucleosome histones and alter the electrostatic properties of chromatin to repress gene expression [47, 48]. HDAC inhibition improves diabetic complications [49]. Total inhibition of HDACs, particularly that of class I HDACs, is associated with endothelial dysfunction, which is an important factor in the pathogenesis of vascular changes [2, 50, 51]. This is accompanied by the production of ROS, which play a causative role in the development and progression of diabetic vascular complications. HDAC3, a class I HDAC that is present in the nucleus and cytoplasm of cells [52], is involved in tumor development, DM, inflammation, and cardiovascular and neurodegenerative diseases [29]. The mechanism of HDAC3 inhibition in anti-inflammation, involves the removal of inhibitory NF-κB p65 acetylation at K122, 123, 314, and 315 [53]. Moreover, HDAC3 deletion induces cardiac hypertrophy, and global deletion of HDAC3 results in embryonic lethality at E9.5 in the heart [52], whereas its inhibition prevents type 1 diabetic cardiomyopathy via epigenetic regulation of the DUSP5–ERK1/2 pathway [54]. Jung and Seong demonstrated that knockdown of endogenous HDAC3 in endothelial cells increases lysine acetylation of endogenous eNOS [55]. The increase in lysine acetylation of eNOS increases NO production by endothelial cells in which HDAC3 is knocked down without affecting eNOS expression. However, HDAC3 also plays a non-deacetylase role in the occurrence and development of diseases. Loss of HDAC3 function protects β-cells from cytokine-induced apoptosis and maintains proper glucose-stimulated insulin secretion [56–58]. Inhibition of HDAC3 in diabetic mice ameliorates diabetes-induced liver damage, and reduces oxidative stress and aortic injury by activating Nrf2 signaling [59]. Increased HDAC3 activity or levels in patients with T2DM is correlated with inflammatory marker expression, poor glycemic control, and insulin resistance [60]. HDAC3 expression is involved in the differentiation of embryonic stem cells into endothelial progenitors; it is critical for endothelial cell survival and as a pro-survival molecule [61–63]. Therefore, we hypothesized that HDAC3 might be a therapeutic target for the treatment of endothelial damage.

In this study, we investigated the effect of HDAC3 inhibition on vascular endothelial function using db/db mice and cultured HUVECs. The results can be summarized as follows: (1) HDAC3 activity was higher in the thoracic aortic vascular endothelium of db/db mice than in that of control mice; (2) HDAC3 inhibition may prevent T2DM-triggered endothelial dysfunction including excessive apoptosis, sprouting, and angiogenesis loss in vivo and in vitro; (3) the protective effect of HDAC3 inhibition on the endothelium is mediated by the activation of Nrf2 signaling, which involves decreasing the Keap1–Nrf2 interaction by reducing Keap1 level; 4) HDAC3 inhibition elicits Nrf2 signaling in association with Nox4 downregulation in the T2DM endothelium; siRNA-mediated Nox4 silencing prevents endothelial dysfunction and activates Nrf2 signaling, defining a Nrf2–Nox4 negative feedback loop. The present results provide insight into the role of HDAC3 in the development of diabetic endothelial dysfunction. However, because other HDAC inhibitors, especially HDAC class I inhibitors, have a protective effect on the cardiovascular system [64–66], the involvement of other HDACs cannot be excluded.

In the resting cell, Nrf2 is sequestered in the cytoplasm by Keap1 and continuously targeted for ubiquitination [67]. Modification of critical cysteine residues in Keap1 leads to the dissociation of the Keap1–Nrf2 complex or increased Nrf2 stability [68]. Civantos and Zhang investigated the association between diabetes-induced liver damage or nephropathy, and increased Nrf2 activity and miR-200a upregulation [59, 69]. However, the relationship between Keap1, Nrf2, and miR-200a in the diabetes-induced endothelium remains unclear and will be further explored in the future.

HDAC3 inhibition decreases Nox4 transcription despite the open chromatin structure in human endothelial cells, thereby improving endothelial function. This may be attributed to HDAC inhibition in endothelial cells associated with increased chromatin accessibility in the human Nox4 promoter region without significant changes in DNA methylation [30]. The present data on the role of HDAC3 in endothelial cells support the role of HDAC3 inhibition on promoting an anti-inflammatory phenotype by altering Nox4 transcriptional levels. Evidence supports the important role of Nox4 in redox signaling. Elsherbiny indicated that doxorubicin‐induced renal damage involves a redox imbalance caused by Nox4 upregulation and Nrf2 downregulation [70]. Hecker confirmed the effect of ROS‐induced Nox4–Nrf2 redox imbalance in persistent lung fibrosis [71]. In addition, inhibitor of NAPDH oxidase, DPI, downregulates Nox4 expression and promotes sliver nanoparticles-induced dysfunction by upregulating Nrf2 [21]. This is consistent with the present results showing that si-*Nox4* upregulated Nrf2 and activated Nrf2 signaling (Fig. 5j and p). Nox4–Nrf2 redox imbalance is thus an important factor for maintaining endothelial function, and restoring balance could be a potential therapeutic strategy.

## Conclusions

In this study, we showed that HDAC3 inhibition decreases T2DM-induced endothelial dysfunction, thereby improving T2DM-induced injury in the vascular endothelium. This protective effect can be partly attributed to the activation of Nrf2 signaling through the suppression of Keap1 level, modulation of the Nox4–Nrf2 redox imbalance, and inhibition of eNOS uncoupling. Keap1 expression and Nox4 transcription were increased in the T2DM endothelium, and HDAC3 inhibition reversed these effects, thereby protecting the endothelium in a Nrf2-dependent manner. The precise mechanism underlying the effect of HDAC3 inhibition on the modulation of Keap1 and Nox4–Nrf2 remains to be elucidated. Future studies should investigate the potential of HDAC3 as an epigenetic regulator in diabetes-associated vasculopathy.

## Supplementary Information

**Additional file 1: Supplementary Figure 1**. HDAC3 inhibition decreases the HG-PA-induced eNOS uncoupling in vitro and the verification of Nrf2 KO in mice.

## Data Availability

All data generated in this study are included in the manuscript.

## Abbreviations

T2DM: Type 2 diabetes mellitus
DV: Diabetic vasculopathy
NG: Normal glucose
HG-PA: High glucose-palmitic acid
Nrf2: Nuclear factor (erythroid-derived 2)-like 2
eNOS: Endothelial nitric oxide synthase
NO: Nitric oxide
L-NAME: NG-nitro-L-arginine methyl ester
Keap1: Kelch-like ECH-associated protein 1
NF-κB: Nuclear factor-κB
Nox4: NAPDH oxidase 4
SFN: Sulforaphane
ARE: Antioxidant response element
IL-1β, 6, 8: Interleukins-1β, 6, 8
TNF-α: Tumor necrosis factor-alpha
VCAM-1: Vascular cell adhesion molecular-1, ICAM-1: intracellular adhesion molecular-1
NQO: NAD(P)H dehydrogenase quinone
SOD2: Superoxide dismutase 2
HO1: Heme oxygenase 1
CAT: Catalase
HDAC: Histone deacetylases
HUVECs: Human umbilical vein endothelial cells
DHE: Dihydroethidium
EBM: Endothelial basal medium
EGM-2: Endothelial cell growth medium-2
NAC: N-Acetyl-L-cysteine
si-*HDAC3*: HDAC3 siRNA
si-*Nrf2*: Nrf2 siRNA
si-*Nox4*: Nox4 siRNA
MAN: Mannitol
qRT-PCR: Quantitative real time-polymerase chain reaction
BSA: Bovine serum albumin
ROS: Reactive oxygen species
HFD: High fat diet
STZ: Streptozotocin
