# Supplementary material for: Histone deacetylase 3 inhibition alleviates type 2 diabetes mellitus-induced endothelial dysfunction via Nrf2
Source: Cell Commun Signal. 2021 Mar 18;19:35. doi: 10.1186/s12964-020-00681-z (PMC7977318; doi:10.1186/s12964-020-00681-z)
Supplement: Supplementary file 2 — Additional file 1: Supplementary Figure 1. HDAC3 inhibition decreases the HG-PA-induced eNOS uncoupling in vitro and the verification of Nrf2 KO in mice. [file 12964_2020_681_MOESM2_ESM.docx]

**Supplementary table1. Details of the primers used in the RT-PCR analysis**

| Primer | Forward | Reverse |
| --- | --- | --- |
| *NQO1*  *NQO2*  *HO1*  *SOD2*  *CAT*  *IL1B*  *IL6*  *IL8*  *TNFα*  *ICAM-1*  *VCAM-1*  *Keap1*  *Nox4*  *GAPDH* | GAAGAGCACTGATCGTACTGGC  GTACTCATTGTCTATGCACACCA  GCCCCAGGATTTGTCAGAGG  GTCACCGAGGAGAAGTACCAGGAGGCG  CCTTCTTGTTCAGGATGTGGTTTTC  TGGGAGTAGATAAGGTACAGCCC  ACTCACCTCTTCAGAACGAATTG  GCCAACACAGAAATTATTGTAAAGCTT  CATCTTCTCAAAACTCGAGTGACAA  TCTTCCTCGGCCTTCCCATA  CCCTTGACCGGCTGGAGATT  CAACCGACAACCAAGACCCC  TGGCTGCCCATC TGGTGAATG  ATGGTGAAGGTCGGTGTGAA | GGATACTGAAAGTTCGCAGGG  TGCCTGCTCAGTTCATCTACA  GGAGGCCATCACCAGCTTGAA  CGCCTCCTGGTACTTCTCCTCGGTGAC  CATGTGTGACCTCAAAGTAGCCAAA  GTAGTGGTGGTCGGAGATTCG  CCATCTTTGGAAGGTTCAGGTTG  AATTCTCAGCCCTCTTCAAAAACTT  TGGGAGTAGATAAGGTACAGCCC  AGGTACCATGGCCCCAAATG  CTGGGGGCAACATTGACATAAAGTG  TCAGTGGAGGCGTACATCAC  CAGCAGCCCTCCTG AAACATGC  TGGAAGATGGTGATGGGCTT |


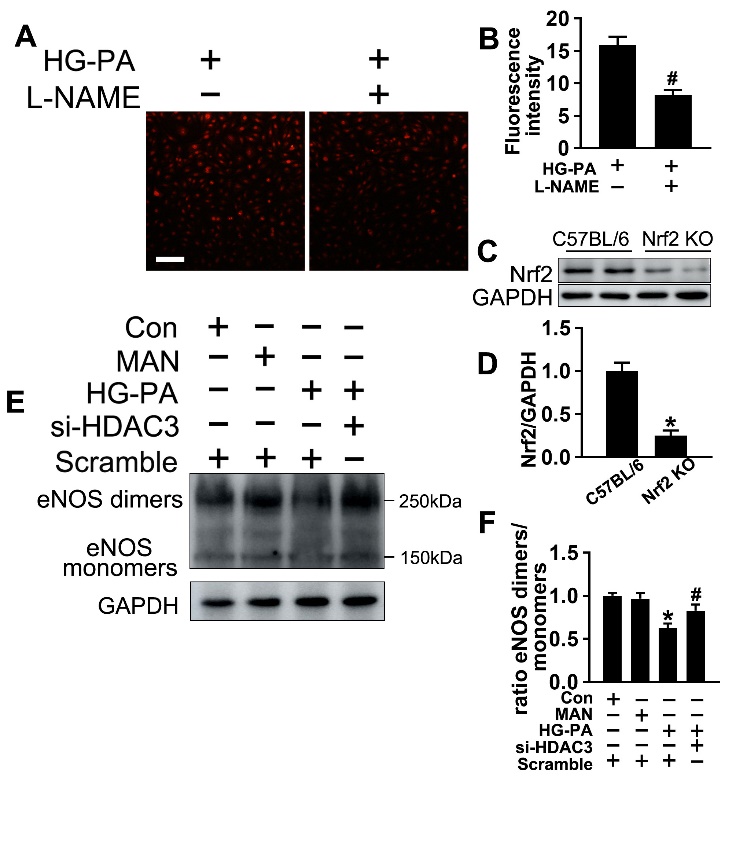


**Supplementary Figure 1**. **A** Superoxide was determined with DHE. Scale bars: 100 μm. **B** The quantitative analysis of fluorescent intensity in (**A**). **C** and **D** The immunoblotting and quantitative analysis of Nrf2 protein level relative to GAPDH protein levels in the the endothelial cells isolated from C57BL/6 mice or Nrf2 KO mice. Values displayed are means ± SEM (n = 5). Significance (**D**): * *P* < 0.05 vs. C57BL/6 mice. **E** The ratio of eNOS dimer/monomers in HUVECs was detected by immunoblotting. **F** The quantitative analysis of the ratio of eNOS dimer/monomers immunoblotting, values displayed are means ± SEM (n = 3). Significance (**F**): * *P* < 0.05 vs. Con or MAN in scrambled HUVECs; # *P* < 0.05 vs. HG-PA in scrambled HUVECs. All above results in graphs were normalized to the first group.
